# Supplementary material for: Content-rich biological network constructed by mining PubMed abstracts
Source: BMC Bioinformatics. 2004 Oct 8;5:147. doi: 10.1186/1471-2105-5-147 (PMC528731; doi:10.1186/1471-2105-5-147)
Supplement: Additional File 5 — The original Chilibot query results of the term "long-term potentiation (LTP)" and 22 other terms, limiting the latest references analyzed to the years 1990, 1995, 2000, and 2004. [file 1471-2105-5-147-S5.bz2 › chilibotAdditionalFile5/ltp1995/html/ACTININ_SYNAPTOPHYSIN.html]

 


 **ACTININ** and **SYNAPTOPHYSIN** 
  
Found 1 abstracts in PubMed,  **1 abstracts were retrieved and analyzed**.  


---

 Search Google  |
 PDF files only 
|  EDU domain only 

---

**Interactive relationship** (e.g. stimulation, inhibition, etc)

**Parallel relationship** (e.g. studied together, co-existance, homology, etc.)

- The present study is the first consecutive analysis of the adult normal human organ of Corti and vestibular hair cells with regard to the expression of F actin, actin associated proteins alpha  **actinin** , alpha and beta spectrins, vinculin and tropomyosin, beta tubulin and the calcium binding protein  **synaptophysin** .  Ref: 7731659 ORL J Otorhinolaryngol Relat SpecORL J Otorhinolaryngol Relat Spec, 1994
